# Work–life balance and work engagement across the European workforce: a comparative analysis of welfare states

**DOI:** 10.1093/eurpub/ckad046

**Published:** 2023-03-23

**Authors:** Janina M Björk-Fant, Pernilla Bolander, Anna K Forsman

**Affiliations:** Department of Developmental Psychology, Faculty of Education and Welfare Studies, Åbo Akademi University, Vaasa, Finland; Department of Management and Organization, Stockholm School of Economics, Stockholm, Sweden; Department of Health Sciences, Faculty of Education and Welfare Studies, Åbo Akademi University, Vaasa, Finland

## Abstract

**Background:**

Few large-scale, comparative studies have examined both the positive mental well-being outcomes of work–life balance and the broader socio-economic context by which it is shaped. Therefore, the aim of this study was to investigate the association between work–life balance and work engagement across a wide range of European welfare states, as well as to examine whether work*–*life balance varies across European countries and whether this variance can be explained by welfare regime, controlling for individual-level factors.

**Methods:**

This study utilized data from the 2015 European Working Conditions Survey. In total, 35 401 workers from 30 European countries could be classified into the adopted welfare regime typology. Work engagement was measured using an ultra-short version of the Utrecht Work Engagement Scale, and work–life balance with a question on the fit between working hours and family or social commitments. Due to the hierarchical structure of the data, multilevel regression models were applied.

**Results:**

A statistically significant positive association between work–life balance and work engagement across the European workforce was found. Between-country variance in work–life balance was demonstrated and this can in part be explained by welfare regime.

**Conclusions:**

While it has long been recognized that occupational stress and work-related mental health problems are shaped by the socio-economic context and thus regarded as public health concerns in Europe, our results suggest that this applies to well-being at work and related support factors as well.

## Introduction

Health, including mental health, is created in peoples’ everyday settings,^1^ and is promoted by empowering them to participate in decisions relevant to their lives and well-being so that they can influence both individual and environmental health determinants.^1^^,^^2^ According to the Social Determinants of Health Framework,^3^ fair employment and decent work are important social determinants of health. Thus, the workplace offers an ideal setting to support mental health promotion of a large share of the population, not only that of workers but also of their families and communities, as well as of wider society.^4^ Occupational stress and work-related mental health problems have long been recognized as public health concerns in Europe,^5^ due to the associated societal consequences in terms of, e.g. absenteeism, labour turnover and disability pension costs.^6^ However, to address well-being and productivity across the European workforce effectively, it has been argued that the traditional focus of occupational safety and health must be expanded to include an understanding and assessment of those factors that lead to healthy, satisfied and productive workers.^7^ Likewise, the WHO Commission on the Social Determinants of Health has advocated a proactive approach to the improvement of working conditions. In this framework, the psychosocial work environment has been highlighted as a key component of working conditions.^3^ In line with this, workplace health promotion has reoriented from an emphasis on wellness activities directed towards the individual, to collective endeavours involving both workers and management to create health-promoting workplaces. This settings approach is holistic and integrative, addressing both individual risk factors and broader organizational and environmental issues.^4^

According to the settings approach,^8^ the core activity of the setting must be considered to make health promotion effective. Studying mental well-being at work thus requires considering how the health determinant of interest relates to productivity, the core activity of organizations.^9^^,^^10^ Although rarely studied in public health research, prior studies in occupational health psychology and related fields have shown that work engagement, defined as a positive, psychological state consisting of the three subcategories vigour, dedication and absorption,^11^ is a particularly important mental well-being outcome to consider in a workplace setting. High levels of work engagement are associated not only with productivity, in terms of financial return^12^ and improved job performance,^13^ but also with important health-related consequences, such as reduced burnout, anxiety and depression.^14^^,^^15^

Another work-related factor that is associated with work engagement is work–life balance. Work–life balance continues to be a policy priority within the European Union.^16^ Work–life balance refers to the ‘overall interrole assessment of compatibility between work and family roles’ (p. 703).^17^ Prior work on work–life balance in European public health research has primarily studied work–life imbalance, e.g. associating it with health problems^18^^,^^19^ and reduced work ability.^20^ However, occupational health psychology and related fields have directed research attention to the association between work–life balance and positive mental well-being outcomes. For example, it has been demonstrated that work–life balance is associated with work engagement, and this association constitutes an area of special interest in the workplace setting. However, results thus far are inconclusive regarding whether this association is negative or positive, since the studies are based on single countries or single organizations using small samples.^21^ Those demonstrating a negative association have explained it by adopting a role strain perspective, in which work and family demands are regarded incompatible due to limited time and energy,^22^ suggesting that engagement in one role requires disengagement in another.^23^ Conversely, those demonstrating a positive association have explained it by adopting a role enrichment perspective, in which positive experiences and affect in one role are regarded to increase engagement in others.^24^^,^^25^

Various demographic and work-related factors have been associated with work–life balance.^26^ Moreover, a growing body of evidence demonstrates that work–life balance is shaped by the wider economic, cultural and political context.^27^^,^^28^ A few comparative studies on the socio-economic factors of work–life balance exist, demonstrating country variation. However, these tend to be focused on identifying factors that can explain work–life imbalance rather than work–life balance. A likely determinant of the cross-country variation in work–life balance is the comprehensiveness and implementation of family–friendly policies in different welfare regimes.^29^ A welfare regime constitutes welfare states with similar political traditions and comprehensiveness in welfare provision.^27^ According to Ferrera^30^ and Bambra and Eikemo’s^27^ classification, the European context includes five welfare regimes: Nordic, Conservative, Liberals, Southern Europe and Central and Eastern Europe (CEE). A prior study has shown that welfare regimes with the most extensive family–friendly policies report the highest levels of work–life balance.^29^ Furthermore, a few studies have demonstrated differences in the association between work–life imbalance and health problems, in terms of poor mental well-being and poor self-related health, across European welfare regimes.^28^^,^^31^

Different welfare regimes approach work–life balance-related policies in distinct ways. In the Nordics, where employment is heavily regulated and the dual-earner model dominates, the state facilitates work–life balance through generous and universal measures such as publicly funded child and elderly services and paid parental leave.^29^^,^^32^ In contrast, in both the Liberals and the Conservatives, families are responsible for finding own solutions to reconcile work and family demands, which often results in men being the main breadwinners and women engaging in part-time work.^33^ In the Liberals, market-based solutions dominate (e.g. childcare provided by private ventures),^34^^,^^35^ whereas family-based support dominates in the Conservatives. Moreover, employment is less regulated in the Liberals than in the Conservatives.^28^ As in the Conservatives, social support provision in the Southern Europe is family-based^27^^,^^35^ and men engage in full-time work; however, they diverge from the Conservatives in that women often engage in full-time childcare. As in the Nordics, a dual-earner model dominates in the CEE; however, employment is weakly regulated and there are traditional gender roles in housework.^31^ The bottom line is that welfare states to a varying degree support health and well-being by redistributing resources between members of society to reduce social exclusion, referred to as the ‘Robin Hood function’, and by redistributing individual resources across the lifespan to insure against social risks, referred to as the ‘Piggy Bank function’.^36^ How these two functions influence work–life balance is an interesting area of investigation, where potential differences between welfare regimes are of particular interest.

However, prior cross-country research on both the positive mental well-being outcomes of work–life balance and the broader socio-economic context by which it is shaped is inconclusive, not least because most existing studies are based on a small number of countries with contradicting results. Even though previous country-level studies have demonstrated an association between work–life balance and work engagement, it remains unexplored whether this association can be found across a wide range of European countries while accounting for the multilevel structure of a large-scale data. This is of relevance to public health research and practice, especially to the design and implementation of family–friendly policies and the creation of health-promoting workplaces.

Against this background, the aim of this study was to investigate the association between work–life balance and work engagement across a wide range of European welfare states, as well as to examine whether work–life balance varies across European countries and whether this variance can be explained by welfare regime, controlling for individual-level factors.

## Methods

### Study sample

The current study was based on data from the 2015 European Working Conditions Survey (EWCS), an interview survey that is conducted by Eurofound on a regular basis.^37^ The 2015 EWCS targeted participants from 35 countries who were identified as workers aged 15 or above. A multi-stage, stratified, random sample approach was employed in each country. There was substantial sample variation across countries depending on the size of the country’s workforce. However, a sample size of minimum 1000 was aimed at with regard to all countries. Countries were also given the opportunity to top-up their sample size (taken up by, e.g. Belgium and Spain). The interviews were conducted face-to-face and via telephone. The number of participants in the 2015 EWCS was 43 850, giving an overall response rate of 42.5%. However, the response rate varied considerably by country (ranging from 11% in Sweden to 78% in Albania). An important reason for the low response rate among countries found at the bottom of the response rate ranking is the two-phase approach, in which respondents were recruited via telephone for a face-to-face interview. The inclusion criteria of the current study specified that respondents were currently workers (i.e. individuals who were unemployed, retired, on leave, full-time homemakers, full-time students, disabled and other were excluded) and could be classified into the five welfare regimes, resulting in a subsample of *N *=* *35 401. Details on the survey can be found elsewhere.^38^

### Measurement

Work engagement was measured using an ultra-short version of the Utrecht Work Engagement Scale (UWES). The mean scale was computed based on the three following items: ‘At my work I feel full of energy (vigour)’, ‘I am enthusiastic about my job (dedication)’ and ‘Time flies when I am working (absorption)’. Responses were scored on a 5-point Likert scale where higher scores indicated higher work engagement. Cronbach’s *α* was 0.73 for the current subsample. Work–life balance was measured using a single-item: ‘How well do your working hours fit in with your family or social commitments?’. Answers were scored on a Likert scale, ranging from 1 (not at all well) to 4 (very well). However, these were dichotomized into very well and less than very well (not at all well, not well and well).

Countries were grouped according to Ferrera^30^ and Bambra and Eikemo’s^27^ classification of welfare typologies (30 countries and 5 regime types in total): Nordic (Sweden, Denmark, Finland and Norway), Conservative (Austria, Belgium, France, Germany, The Netherlands, Luxembourg and Switzerland), Liberals (UK and Ireland), Southern Europe (Greece, Spain, Italy, Portugal, Cyprus and Malta) and CEE (Estonia, Lithuania, Hungary, Czech Republic, Poland, Latvia, Romania, Slovakia, Slovenia, Bulgaria and Croatia). Gender (man, woman) was included as a dichotomous variable. Age (in years) was included as a continuous variable. Dichotomous control variables were cohabiting partner (yes, no), cohabiting children (yes, no), supervisory position (yes, no), employment status (full-time, part-time), International Standard Classification of Education (low ≤ 4, high ≥ 5) and whether the respondent was the most significant contributor to the household income (yes, no).

### Statistical analyses

SPSS version 27 (SPSS, SPSS IBM Statistics, USA) statistical package was employed to perform the statistical analyses. Initially, a missing data analysis was conducted followed by descriptive statistics to present sample characteristics. Given the multilevel structure of the data, we applied multilevel regression analyses with individuals (level 1) nested within countries (level 2). By applying random intercept multilevel models, between-country variation can be studied. All multilevel models included a fixed part and a random component.^39^^,^^40^ First, multilevel linear regression analysis was applied to examine the association between work–life balance and work engagement. As an initial step, the random intercept model was built to estimate the between-country variation of the intercept. The intraclass correlation coefficient (ICC) was calculated to estimate the proportion of the variance accounted for by clustering. Further, the Design EFFect (DEFF) was calculated which takes both the mean cluster size (*N*) and within-cluster homogeneity (ICC) into account to quantify the degree to which a multilevel sample differs from a one-level random sample^39^ (see also Supplementary material S1). In the second step, work engagement was entered together with the control variables. Estimate values with 95% confidence intervals are presented. Next, multilevel logistic regression analysis was applied to examine the variation of work–life balance between countries and the underlying factors for this variation. This latter analysis constituted three models. The random intercept model was run to estimate the between-country variation of the intercept and the ICC^40^ (see also Supplementary material S1). The control factors were added in Model 2 and welfare regimes in Model 3. Model fit statistics are presented [−2 log-likelihood, Akaike information criterion (AIC) and Bayesian information criterion (BIC)).

## Results

Study sample characteristics are presented in Table 1. Women slightly more often than men reported a good work–life balance. In general, women also tended to report higher work engagement scores.

**Table 1:** Overview of the study sample according to variables measuring work engagement, work–life balance and control factors by gender and in total [*N *=* *35 401; *N* (%) or mean (SD)]

	Men	Women	Total
	*N* = 17 498 (49.4)	*N* = 17 897 (50.6)	*N* = 35 401 (100)
Work engagement	3.94 (0.71)	3.96 (0.70)	3.95 (0.70)
Work–life balance			
Very well	4861 (27.9)	5668 (31.8)	10 529 (29.9)
Less than very well	12 558 (72.1)	12 179 (68.2)	24 737 (70.1)
Age	43.90 (12.86)	43.66 (12.37)	43.49 (11.95)
Educational level			
High	5267 (30.2)	6744 (37.8)	12 011 (34.1)
Low	12 153 (69.8)	11 101 (62.2)	23 254 (65.9)
Cohabiting partner			
Yes	11 751 (67.2)	11 408 (63.8)	23 159 (65.5)
No	5736 (32.8)	6483 (36.2)	12 219 (34.5)
Cohabiting children			
Yes	7526 (43.0)	9025 (50.4)	16 551 (46.8)
No	9967 (57.0)	8865 (49.6)	18 832 (53.2)
Most significant contributor to the household income			
Yes	14 071 (85.6)	8396 (51.3)	22 467 (68.5)
No	2364 (14.4)	7957 (48.7)	10 321 (31.5)
Employment status			
Full-time	15 350 (88)	12 748 (71.4)	28 098 (79.6)
Part-time	2101 (12)	5106 (28.6)	7207 (20.4)
Supervisory position			
Yes	3579 (20.7)	2202 (12.4)	5781 (16.5)
No	13 708 (79.3)	15 516 (87.6)	29 224 (83.5)
Welfare regime			
Nordic	1850 (10.6)	1873 (10.5)	3723 (10.5)
Conservative	4767 (27.2)	4827 (27.0)	9594 (27.1)
Liberals	1407 (8.0)	1215 (6.8)	2622 (7.4)
Southern Europe	4406 (25.2)	4031 (22.5)	8437 (23.8)
CEE	5068 (29.0)	5951 (33.3)	11 019 (31.1)

Results of multilevel regression analyses are presented by gender and in total. Analysing the association between work–life balance and work engagement (dependent variable) using multilevel linear regression, the results of the random intercept model showed that multilevel analysis was warranted (between-country variance was 0.02 and ICC was 0.04 for both men and women, and DEFF was 21.68 for men and 25.01 for women), random intercept model not presented. In Table 2, a positive association between work–life balance and work engagement is shown. Separate analyses for men and women reveal only marginal differences, showing that the association is slightly stronger among men than among women.

**Table 2:** Association between work–life balance and work engagement by gender and in total: Results of multilevel linear regression (estimate and 95% confidence intervals) (total *N* = 35 401; Men *N* = 17 498; Women *N* = 17 897)

	Work engagement
	Estimate (95 % CI)
Total	
Fixed effect: Work–life balance	0.27 (0.26–0.29)
Random effects	
Between-country variance	0.01
ICC	0.03
DEFF	31.90
Men	
Fixed effect: Work–life balance	0.28 (0.25–0.30)
Random effects	
Between-country variance	0.01
ICC	0.03
DEFF	16.58
Women	
Fixed effect: Work–life balance	0.27 (0.25–0.30)
Random effects	
Between-country variance	0.02
ICC	0.03
DEFF	18.50

*Notes*: Results are adjusted for control factors (i.e. age, educational level, cohabiting partner and children, employment status and supervisory position).

Table 3 shows results from multilevel logistic regression analyses with work–life balance as the dependent variable. The results of the random intercept model warranted multilevel analysis. Significant between-country variation was observed with an ICC of 0.04 for men and 0.05 for women. Further, the between-country variance was higher for women than for men. Individual-level variables were added in Model 2. No substantial reduction in the between-country variance was found for men nor for women when these variables were included. In Model 3, welfare regime was added. The inclusion of this variable yielded substantial reduction of the between-country variance for both men and women. Moreover, Supplementary table S1 shows that working men in both Southern Europe and CEE were significantly less likely to report work–life balance than working men in the Nordics, while no statistically significant difference was found between workers in Conservative and Liberal welfare regimes and workers in the Nordic welfare regime. For women, Southern Europe was the only welfare regime in which workers had significantly lower odds of reporting work–life balance compared with workers in the Nordics.

**Table 3:** Multilevel logistic regression analysis: reduction in the between-country differences in work–life balance (total *N *=* *35 401; men *N *=* *17 498; women *N *=* *17 897)

	Work–life balance		
	Model 1:	Model 2:	Model 3:
	Random intercept	M1 + Control factors	M2 + Welfare regime
Total			
Random effects			
Country level			
Between-country variance	0.16	0.16	0.12
ICC	0.05	0.05	0.03
Statistics			
−2 Log-likelihood	156 702.941	143 364.424	143 380.42
AIC	156 704.941	143 366.425	143 382.42
BIC	156 713.412	143 374.8	143 390.795
Men			
Random effects			
Country level			
Between-country variance	0.14	0.13	0.09
ICC	0.04	0.04	0.02
Statistics			
−2 Log-likelihood	78 004.829	72 184.32	72 201.112
AIC	78 006.83	72 186.32	72 203.112
BIC	78 014.595	72 194.001	72 210.793
Women			
Random effects			
Country level			
Between-country variance	0.19	0.18	0.15
ICC	0.05	0.05	0.04
Statistics			
−2 Log-likelihood	78 776.697	71 214.873	71 231.51
AIC	78 778.697	71 216.874	71 233.51
BIC	78 786.486	71 224.555	71 241.191

## Discussion

Overall, the study’s findings provided support for a statistically significant positive association between work–life balance and work engagement across a wide variety of European welfare states, adjusting for individual-level control factors. This lends support to the role enrichment perspective and extends the findings of prior small and single-country sample studies.^24^^,^^25^

The present study adds to prior, large-scale studies on mental health and well-being across European welfare states^28^^,^^31^ by expanding the traditional focus on risk factors to include support factors.^5^^,^^7^ That is, while both Lunau *et al*.^28^ and Mensah and Adjei ^31^ have demonstrated that work–life imbalance can be associated with health problems, such as poor mental well-being, the present study shows that work–life balance, in turn, can be associated with positive aspects of mental well-being at work, such as work engagement.

Further, the current study addressed the pressing need to account for the wider cultural and political context in the study of well-being at work and work–life balance in particular.^28^ Applying multilevel modelling, between-country variance in work–life balance was demonstrated for both men and women and the variance was higher for women. While the between-country variance was not substantially reduced for men nor for women by including individual-level control variables, the variations between countries were substantially reduced for both men and women when welfare regime was included.

Analysing the association between welfare regime and work–life balance, our expectation was that the Nordic welfare regime would stand out from the others in good terms as it usually is referred to as a good example when it comes to the promotion of work–life balance.^29^ Indeed, results demonstrated that both men and women in the Southern European welfare regime and men in the CEE welfare regime were less likely to report work–life balance when compared with men and women in the Nordic welfare regime. With regard to Southern Europe, the family-based social support with a clear division of men engaging in full-time work and women in full-time childcare is likely to explain why workers in this welfare regime were less likely to report work–life balance.^27^^,^^35^ With regard to CEE, where a dual-earner model dominates and there are traditional gender roles in housework, it was unexpected that men, not women, were less likely to report work–life balance compared with their Nordic counterparts. It is possible that the weakly regulated labour market in CEE is part of the explanation to why this finding was only found among working men.^31^ However, workers in the other welfare regimes (both men and women) did not significantly differ from those in the Nordic.

The present, large-scale survey study based on 2015 EWCS data was the first comparative study to apply multilevel modelling in the analysis of the association between work–life balance and work engagement. Further, it contributed to the relatively small but growing literature on how work–life balance is shaped by the socio-economic context, demonstrating that between-country variance exists, and that welfare regime can explain part of this variance.

The study has certain strengths and limitations. First, causal relationships could not be determined between the variables as the study used cross-sectional data. For example, it is possible that high levels of work engagement can help individuals to balance their work and personal life, rather than the other way around. Considerable variation in the response rate between the countries could be associated with bias. However, there were no to only marginal changes in the results when we ran additional multilevel analyses in which we adjusted for response rate (results not shown). Given the large sample and the hierarchical structure of the data, a strength of this study was the use of multilevel modelling.

Furthermore, work engagement was measured using an ultra-short version of the well-validated UWES-scale, while work–life balance was measured using a one-item statement. However, single-item statements about work–life balance have been validated in a previous study, demonstrating that single-item statements about work–life balance can be regarded acceptable and even useful due to practical constraints.^41^

The adopted welfare regime typology highlighted how work–life balance is shaped by the socio-economic context, although not specifically developed to capture work–life balance policies. However, other approaches, such as ones taking institutional and labour market factors as their starting points, would be useful in future studies.

In conclusion, study findings demonstrated that work–life balance and work engagement are associated across European welfare states. Furthermore, the variations between countries in work–life balance were reduced when welfare regime was included in the analysis. Our findings thus suggest that work–life balance in part is shaped by the socio-economic context and this should be considered in the design and implementation of future work–life policies and in the creation of health-promoting workplaces across Europe.

## Supplementary Material

ckad046_Supplementary_Data

## Data Availability

The EWCS datasets are stored with the UK Data Service (UKDS) in Essex, UK and are publicly available via their website (https://ukdataservice.ac.uk/). Users are required to be registered with the UK Data Service. Users who register must accept the End User License (EUL), which is agreed to during the registration process. This study demonstrates a statistically significant positive association between work–life balance and work engagement across European welfare states. There is variance between European countries in work–life balance and this can in part be explained by welfare regime. Working men in Southern Europe and CEE are less likely to report work–life balance than working men in the Nordics, the same holds true for working women but only with regard to those in Southern Europe. Work–life balance is shaped by the socio-economic context and this should be considered in the design and implementation of future work–life policies and in the creation of health-promoting workplaces across Europe. The traditional focus on occupational safety and health in public health research must be expanded to include a focus on those factors that lead to healthy, satisfied and productive workers.
