# Supplementary material for: Work–life balance and work engagement across the European workforce: a comparative analysis of welfare states
Source: Eur J Public Health. 2023 Mar 23;33(3):430–4. doi: 10.1093/eurpub/ckad046 (PMC10234642; doi:10.1093/eurpub/ckad046)
Supplement: ckad046_Supplementary_Data [file ckad046_supplementary_data.docx]

**Supplemental Material 1**

The Intraclass Correlation Coefficient (ICC) was calculated to estimate the proportion of the variance accounted for by clustering. ICC can range from 0 to 1 (where 0 indicates no between-country variation and 1 indicates no within-country variation). It has been demonstrated that an ICC as low as 0.01 can warrant multilevel linear modelling [39, 40].

The Design EFFect (DEFF) takes both the mean cluster size (*N*) and within-cluster homogeneity (ICC) into account to quantify the degree to which a multilevel sample differs from a one-level random sample. The DEFF can range from 1 (no difference) to *N* (a maximal difference), where a DEFF of ≥1.5 warrants multilevel modelling [39].

**Supplemental Table 1**

Supplementary Table 1. Multilevel logistic regression analysis: association between individual-level variables, welfare regime type, and work-life balance (Total *N* = 35401; Men *N* = 17498; Women *N* = 17897; odds ratios and 95 % CI).

|  |  | **Work-life balance** | | | | | | | | |
| --- | --- | --- | --- | --- | --- | --- | --- | --- | --- | --- |
|  |  | **Total** | | | **Men** | | | **Women** | | |
|  |  | **Model 1:**  **Random intercept** | **Model 2:**  **M1 + Control factors** | **Model 3:**  **M2 + Welfare regime** | **Model 1:**  **Random intercept** | **Model 2:**  **M1 + Control factors** | **Model 3:**  **M2 + Welfare regime** | **Model 1:**  **Random intercept** | **Model 2:**  **M1 + Control factors** | **Model 3:**  **M2 + Welfare regime** |
| **Fixed effects** |  |  |  |  |  |  |  |  |  |  |
| Age |  |  | 1.01 (1.01–1.01) | 1.01 (1.01–1.01) |  | 1.01 (1.01–1.02) | 1.01 (1.01–1.02) |  | 1.01 (1.01–1.01) | 1.01 (1.01–1.01) |
| Educational level | Low |  | 1.00 | 1.00 |  | 1.00 | 1.00 |  | 1.00 | 1.00 |
|  | High |  | 1.24 (1.17–1.31) | 1.24 (1.17–1.30) |  | 1.34 (1.24–1.45) | 1.34 (1.24–1.45) |  | 1.15 (1.07–1.24) | 1.15 (1.07–1.24) |
|  |  |  |  |  |  |  |  |  |  |  |
| Cohabiting partner | No |  | 1.00 | 1.00 |  | 1.00 | 1.00 |  | 1.00 | 1.00 |
|  | Yes |  | 0.87 (0.83–0.93) | 0.87 (0.82–0.93) |  | 0.87 (0.79–0.95) | 0.87 (0.79–0.95) |  | 0.89 (0.82–0.97) | 0.89 (0.81–0.97) |
|  |  |  |  |  |  |  |  |  |  |  |
| Cohabiting children | No |  | 1.00 | 1.00 |  | 1.00 | 1.00 |  | 1.00 | 1.00 |
|  | Yes |  | 0.82 (0.76–0.87) | 0.82 (0.77–0.86) |  | 0.81 (0.74–0.88) | 0.81 (0.74–0.88) |  | 0.81 (0.75–0.87) | 0.81 (0.75–0.87) |
|  |  |  |  |  |  |  |  |  |  |  |
| Employment status | Part-time |  | 1.00 | 1.00 |  | 1.00 | 1.00 |  | 1.00 | 1.00 |
|  | Full-time |  | 0.54 (0.51–0.58) | 0.55 (0.51–0.58) |  | 0.62 (0.56–0.69) | 0.62 (0.56–0.69) |  | 0.50 (0.47–0.55) | 0.51 (0.47–0.55) |
|  |  |  |  |  |  |  |  |  |  |  |
| Supervisory position | No |  | 1.00 | 1.00 |  | 1.00 | 1.00 |  | 1.00 | 1.00 |
|  | Yes |  | 0.81 (0.76–0.87) | 0.81 (0.76–0.87) |  | 0.85 (0.78–0.93) | 0.85 (0.78–0.93) |  | 0.76 (0.68–0.84) | 0.75 (0.67–0.84) |
|  |  |  |  |  |  |  |  |  |  |  |
| Most significant contributor to the household income | No |  | 1.00 | 1.00 |  | 1.00 | 1.00 |  | 1.00 | 1.00 |
|  | Yes |  | 0.86 (0.81–0.91) | 0.85 (0.81–0.91) |  | 0.82 (0.74–0.91) | 0.82 (0.74–0.91) |  | 0.89 (0.82–0.97) | 0.89 (0.82–0.97) |
| Welfare regime type |  |  |  |  |  |  |  |  |  |  |
|  | Nordic |  |  | 1.00 |  |  | 1.00 |  |  | 1.00 |
|  | Conservative |  |  | 0.79 (0.51–1.21) |  |  | 0.81 (0.55–1.19) |  |  | 0.75 (0.46–1.22) |
|  | Liberals |  |  | 1.07 (0.59–1.94) |  |  | 0.98 (0.57–1.66) |  |  | 1.19 (0.61–2.33) |
|  | Southern Europe |  |  | 0.53 (0.34–0.83) |  |  | 0.54 (0.36–0.81) |  |  | 0.53 (0.32–0.87) |
|  | CEE |  |  | 0.62 (0.41–0.92) |  |  | 0.59 (0.41–0.85) |  |  | 0.64 (0.41–1.02) |
|  |  |  |  |  |  |  |  |  |  |  |
